# Salinomycin enhances doxorubicin-induced cytotoxicity in multidrug resistant MCF-7/MDR human breast cancer cells via decreased efflux of doxorubicin

**DOI:** 10.3892/mmr.2015.3633

**Published:** 2015-04-16

**Authors:** KWANG-YOUN KIM, SANG-HUN KIM, SUN-NYOUNG YU, SUEL-KI PARK, HYEUN-DEOK CHOI, HAK-SUN YU, JAE-HOON JI, YOUNG-KYO SEO, SOON-CHEOL AHN

**Affiliations:** 1 Department of Microbiology and Immunology, Pusan National University School of Medicine, Yangsan, Gyeongsangnam-do 626-870, Republic of Korea; 2 School of Life Sciences, Ulsan National Institute of Science and Technology, Ulsan 689-798, Republic of Korea; 3 Immunoregulatory Therapeutics Group in Brain Busan 21 Project, Pusan National University School of Medicine, Yangsan, Gyeongsangnam-do 626-870, Republic of Korea; 4 Department of Parasitology, Pusan National University School of Medicine, Yangsan, Gyeongsangnam-do 626-870, Republic of Korea; 5 Genome Instability Research Center, Ajou University School of Medicine, Suwon, Gyeonggi-do 443-721, Republic of Korea

**Keywords:** breast cancer, multidrug resistance, salinomycin, doxorubicin

## Abstract

Salinomycin is a monocarboxylic polyether antibiotic, which is widely used as an anticoccidial agent. The anticancer property of salinomycin has been recognized and is based on its ability to induce apoptosis in human multidrug resistance (MDR). The present study investigated whether salinomycin reverses MDR towards chemotherapeutic agents in doxorubicin-resistant MCF-7/MDR human breast cancer cells. The results demonstrated that doxorubicin-mediated cytotoxicity was significantly enhanced by salinomycin in the MCF-7/MDR cells, and this occurred in a dose-dependent manner. This finding was consistent with subsequent observations made under a confocal microscope, in which the doxorubicin fluorescence signals of the salinomycin-treated cells were higher compared with the cells treated with doxorubicin alone. In addition, flow cytometric analysis revealed that salinomycin significantly increased the net cellular uptake and decreased the efflux of doxorubicin. The expression levels of MDR-1 and MRP-1 were not altered at either the mRNA or protein levels in the cells treated with salinomycin. These results indicated that salinomycin was mediated by its ability to increase the uptake and decrease the efflux of doxorubicin in MCF-7/MDR cells. Salinomycin reversed the resistance of doxorubicin, suggesting that chemotherapy in combination with salinomycin may benefit MDR cancer therapy.

## Introduction

Breast cancer is one of the most frequently diagnosed types of cancer in females (1). The risk factors for breast cancer are age, alcohol consumption, body mass index, hormone replacement therapy and reproductive factors (2). The majority of females with breast cancer develop metastasis in common sites, including the bone, liver and lung (3). Several reports have revealed that males are also susceptible to breast cancer in the United States (4). The progression of breast cancer between an estrogen-dependent, non-metastatic phenotype and an estrogen-independent, invasive, metastatic phenotype are accompanied by chemoresistance (5). Treatment for breast cancer includes several approaches, including surgical and/or pharmacological approaches by estrogenic signaling in estrogen receptor-positive breast cancers (6). However, there is no targeted therapy for estrogen-independent breast cancer (7). Several studies have investigated combination therapies to improve the response to chemotherapy (8,9).

Continuous exposure of cancer cells to drugs leads to the development of a multidrug resistance (MDR) phenotype. The resistance of multiple chemotherapeutic drugs has been recognized as a major contributor to the failure of cancer therapy and obstacle in the successful treatment of numerous types of malignancy (10). MDR occurs through several mechanisms, including increased adenosine triphosphate (ATP)-dependent efflux (11). The classical cellular mechanism of MDR involves efflux of the drug by various membrane transport proteins. ATP-binding cassette (ABC) transporters are a family of proteins, which mediate MDR via ATP-dependent drug efflux pumps (12). Several transport proteins of the ABC superfamily have been characterized and include P-glycoprotein (P-gp, MDR-1; ABCB-1), multidrug resistance-associated protein-1 (MRP-1; ABCC-1) and breast cancer resistance protein (BCRP; ABCG-2) which are overexpressed in chemoresistant cells (13).

Ionophore is a lipid-soluble molecule, which is usually produced by a variety of microbes to transport ions across biological membranes and increase the feeding efficiency of ruminant animals (14,15). Salinomycin is one of the monocarboxylic ionophores isolated from *Streptomyces albus* (16). Salinomycin has been demonstrated to cause the death of breast cancer stem cells (CSCs) more efficiently compared with the anticancer drug, paclitaxel (17). Several reports have suggested that salinomycin induces apoptosis via cell cycle arrest and reactive oxygen species (ROS)-mediated mitochondrial pathways in a diversity of cancer cells (18,19). Salinomycin also triggers apoptosis by overcoming ABC transporter-mediated multidrug and apoptosis resistance in MDR cancer cells (20). Other reports have indicated that salinomycin induces apoptosis through the overexpression of B-cell lymphoma 2 and enhanced proteolytic activity, independent of the p53 tumor suppressor protein in MDR cancer cells (18). Salinomycin-induced activation of autophagy, with concomitant generation of ROS and activation of endoplasmic reticulum stress has also been observed in human cancer cells (21,22). The effect of salinomycin on the death of CSCs and MDR types of cancer may demonstrate a novel class of anticancer agents. In the present study, it was hypothesized that salinomycin may act as a reverser of MDR and benefit patients receiving chemotherapy. The reversal effect of salinomycin was investigated and the underlying mechanism of action was evaluated in human breast cancer doxorubicin-sensitive MCF-7 and doxorubicin-resistant MCF-7/MDR cells.

## Materials and methods

### Reagents and antibodies

Salinomycin, doxorubicin and 3-(4,5-Dimethyl-thiazol-2-yl)-2,5-diphenyl-etrazolium bromide (MTT) were purchased from Sigma-Aldrich (St. Louis, MO, USA). The salinomycin and doxorubicin were dissolved in methanol (Samchun Pure Chemical, Pyeongtaek, Korea) and distilled water as 20 mM stock solutions, respectively. Antibodies for MDR-1, MRP-1 and β-actin were purchased from Enzo Life Sciences (Farmingdale, NY, USA) and Santa Cruz Biotechnology, Inc. (Santa Cruz, CA, USA), respectively. Gat-anti-mouseimmunoglobulin (Ig)G was purchased from Enzo Life Sciences. The ECL Western kit was purchased from GE Healthcare Bio-Sciences (Pittsburgh, PA, USA).

### Cell lines and cell culture

The MCF-7 human breast cancer cells lines were obtained from American Type Culture Collection (Manassas, VA, USA). The MCF-7/MDR cell lines were generated through sequential exposure to increasing concentrations of doxorubicin (0.1–1 *µ*M). The cells were maintained and cultured in Dulbecco’s modified Eagle’s medium (DMEM; WelGENE Inc., Daegu, Republic of Korea), supplemented with 10% fetal bovine serum (FBS; WelGENE Inc.), 100 U/ml penicillin and 100 *µ*g/ml streptomycin (WelGENE Inc.) at a temperature of 37°C in a humidified atmosphere with 5% CO_2_. Doxorubicin (1 *µ*M) was added to the culture medium to maintain the MDR characteristics of the MCF-7/MDR cells.

### Cell viability analysis

The doxorubicin-sensitive MCF-7 and doxorubicin-resistant MCF-7/MDR cells were seeded at a density of 1×10^4^ cells/well into a 6-well plate. The cells were treated with doxorubicin (0.1–20 *µ*M) and salinomycin (0.5–20 *µ*M), either alone or in combination for 72 h. Subsequently, MTT solution (0.5 mg/ml) was added to the culture medium for a further 4 h incubation in a 37°C and 5% CO_2_ atmosphere. The cells were then dissolved in dimethyl sulfoxide (Junsei Chemical Co., Tokyo, Japan). Colorimetric analysis was performed at 570 nm using an ELISA reader (VERSAmax microplate reader; Molecular Devices, Toronto, ON, Canada).

### Intracellular accumulation of doxorubicin

Doxorubicin is a well-known P-gp substrate and is frequently used to treat breast cancer. In the present study, the MCF-7/MDR cells were seeded at a density of 5×10^4^ cells in a glass-bottom dish. The cells were treated with 10 *µ*M doxorubicin, alone or in combination with salinomycin (10–20 *µ*M). Following 3 h incubation at 37°C, the cells were washed three times with phosphate-buffered saline (PBS). To determine changes in the efflux of intracellular doxorubicin, a separate set of samples was incubated for 2 h at 37°C in fresh medium without doxorubicin or salinomycin, as a control. The cells were visualized using a laser scanning confocal microscope (Olympus Fluoview FV1000; Olympus, Tokyo, Japan). To quantify the intracellular accumulation of doxorubicin, the cells were exposed to doxorubicin, alone or in combination with salinomycin for 3 h at 37°C. The control samples were incubated without doxorubicin or salinomycin, or with salinomycin (10–20 *µ*M) alone for 2 h at 37°C. The cells were analyzed using flow cytometry (FACScalibur; Becton Dickinson, Franklin Lakes, NJ, USA) and the accumulation of doxorubicin was calculated using Cell Quest Pro software on Mac^®^OS 9 (Becton Dickinson).

### Reverse transcription-quantitative polymerase chain reaction (RT-qPCR)

The MCF-7/MDR cells were seeded into 60 cm^2^ cell culture dishes (1×10^6^ cells). Following 24 h incubation at 37°C, the cells were treated with salinomycin (10 or 20 *µ*M) for 3 and 6 h. Subsequently, the cells were harvested, and total RNA isolation was performed using a RiboEX_column Total RNA Purification kit (GeneAll, Seoul, Korea). RT-qPCR amplification was performed using primers for MDR1, MRP1 and GAPDH. The PCR primers were designed using the Primer3 programs (http://frodo.wi.mit.edu) and the sequences were as follows: MDR1, forward 5′-ATATCAGCAGCCCACATCAT-3′ and reverse 5′-GAAGCACTGGGATGTCCGGT-3′; MRP1, forward 5′-TGTGAGCTGGTCTCTGCCATA-3′ and reverse 5′-CTGGCTCATGCCTGGACTCT-3′ and GAPDH, forward 5′-GCCAAAAGGGTCATCATCTC-3′ and reverse 5′-GTAGAGGCAGGGATGATGTTC-3′. The PCR cycles were as follows: 94°C for 5 min; 33 cycles at 94°C for 1 min, 58°C for 1 min and 72°C for 1 min; followed by 72°C for 10 min. For The RT-qPCR was performed using low cycle numbers to avoid saturation, in triplicate samples.

### Western blot analysis

The cell extracts were prepared by incubating the cells in lysis buffer, containing 150 mM NaCl, 10 mM Tris (pH 7.4), 5 mM EDTA (pH 8.0), 1% Triton X-100, 1 mM PMSF, 20 mg/ml aprotinin, 50 *µ*g/ml leupeptin, 1 mM benzidine and 1 mg/ml pepstatin. Equal quantities of proteins (40 *µ*g) were electrophoretically separated using sodium dodecyl sulfate-polyacrylamide gel electrophoresis (SDS-PAGE) on an 8% gel, and were then transferred onto a polyvinylidenefluoride (PVDF) membrane (GE Healthcare Bio-Sciences). Following blocking with Tris-buffered saline with Tween 20 (TBS-T) containing 20 mM Tris (pH 7.4), 150 mM NaCl and 0.1% Tween 20, with5% skim milk, the membranes were incubated with primary (MDR-1, MRP-1 and β-actin; 1:1,000; 4°C overnight) and secondary (goat-anti-mouse; 1:5,000, 2 h at room temperature) antibodies. The membranes were then washed with TBS-T buffer and visualized using enhanced chemiluminescence western blotting detection reagents. The density of each band was determined using a fluorescence scanner (LAS 3000; FujiFilm, Tokyo, Japan) and analyzed using Multi Gauge V3.0 software (FujiFilm).

### Statistical analysis

The experiments were repeated at least three times with consistent results. Unless otherwise stated, the data are expressed as the mean ± standard deviation. Analysis of variance was used to compare the experimental groups with the control group, whereas comparisons among multiple groups were performed by Tukey’s multiple comparison test using Graphpad InStat V3.05. P<0.05 was considered to indicate a statistically significant difference.

## Results

### Salinomycin sensitizes MCF-7/MDR cells against doxorubicin

**Figure 1:**
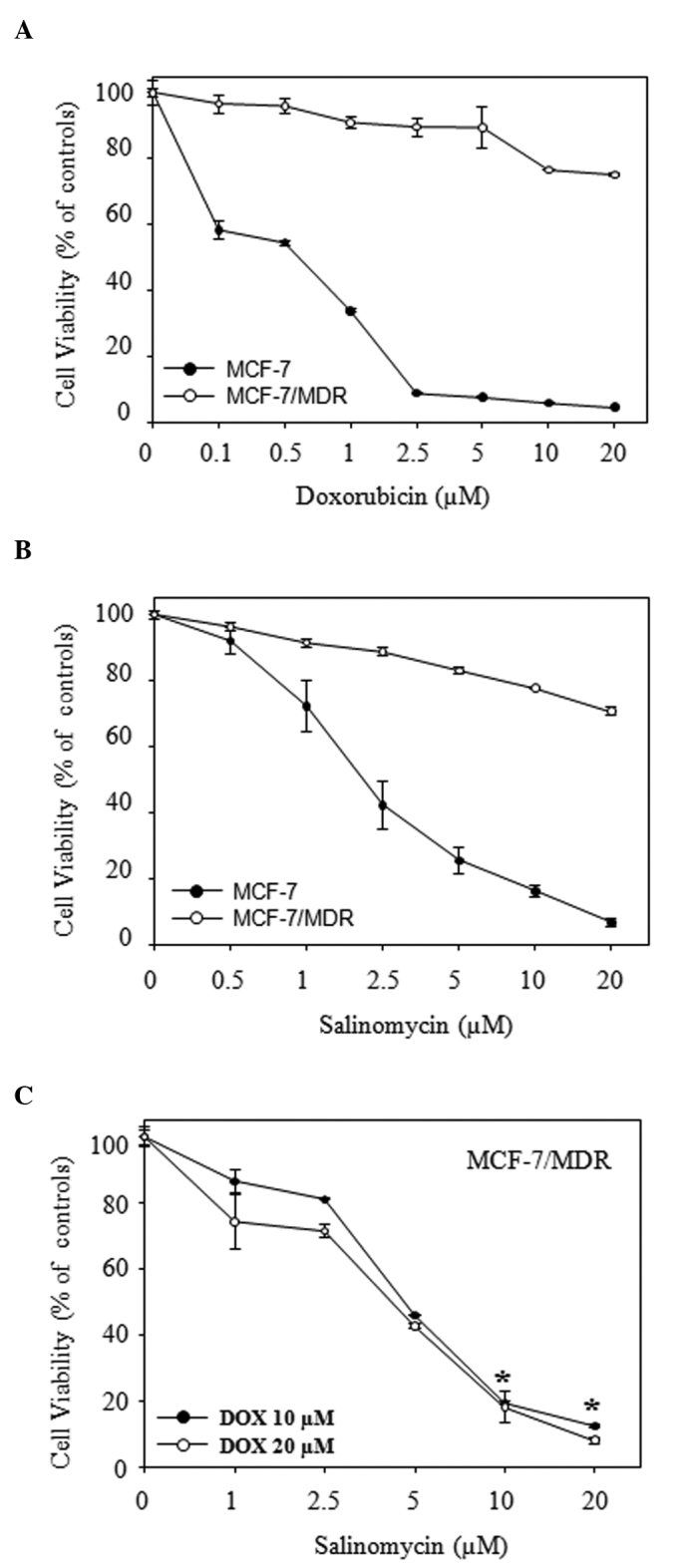
Salinomycin enhances doxorubicin-mediated cytotoxicity in MCF-7/MDR cells. The cells were treated for 72 h. Cell viability was determined using an 3-(4,5-Dimethyl-thiazol-2-yl)-2,5-diphenyl-etrazolium bromide assay. (A) Doxorubicin-treated MCF-7 and MCF-7/MDR cells; (B) salinomycin-treated MCF-7 and MCF7/MDR cells; (C) combined treatment of MCF-7 and MCF7/MDR cells with doxorubicin and salinomycin. All data are representative of at least three times independent experiments. and are expressed as the mean ± standard deviation. ^*^P< 0.05. DOX, doxorubicin; MDR, multidrug resistance.

The effect of salinomycin on cytotoxicity was investigated using an MTT assay on the doxorubicin-sensitivite MCF-7 or doxorubicin-resistant MCF-7/MDR cells. Following treatment with various concentrations of doxorubicin for 72 h, the viability of cells against MCF-7 was reduced in a dose-dependent manner. The half maximal inhibitory concentration of doxorubicin was <1 *µ*M. By contrast, the MCF-7/MDR cells were highly resistant to doxorubicin (Fig. 1A). In addition, salinomycin significantly inhibited cell viability in the MCF-7 cells, however MCF-7/MDR cells exhibited only mild cytotoxicity compared with the MCF-7 cells when the cells were exposed to salinomycin alone, at the levels up to 20 *µ*M (Fig. 1B). Salinomycin significantly enhanced the cytotoxicity of doxorubicin on the MCF-7/MDR cells in the dose-dependent manner (Fig. 1C). In the presence of 10 *µ*M salinomycin, the viabilities of these cells following 72 h treatment with 10 or 20 *µ*M doxorubicin were 19.4 and 18.2%, respectively, However, in the presence of 20 *µ*M salinomycin, the viabilities at the same concentrations of doxorubicin were 12.4 and 8.1%, respectively. No significant effects of salinomycin on doxorubicin cytotoxicity were observed on the MCF-7 cells (data not shown).

### Salinomycin increases doxorubicin accumulation and decreases efflux in MCF-7/MDR cells

**Figure 2:**
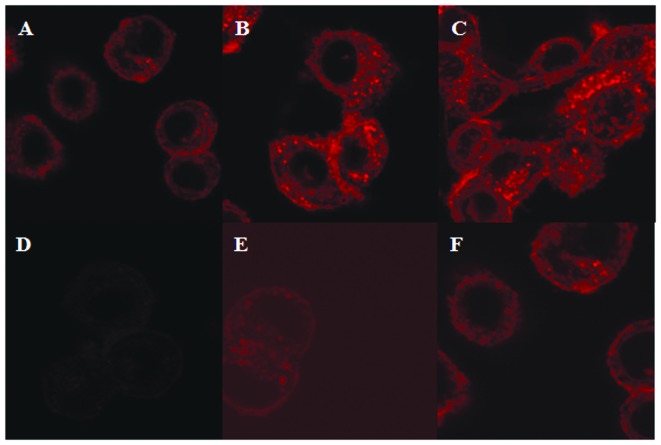
Salinomycin increases the accumulation and decreases the efflux of doxorubicin in MCF-7/MDR cells. (A–C) Confocal images of the accumulations of doxorubicin. The MCF-7/MDR cells were treated doxorubicin in the presence or absence of salinomycin for 3 h. (D–F) Confocal images of decreases of doxorubicin. The MCF-7/MDR cells were removed from doxorubicin and salinomycin treatment further and were observed after 2 h The cells were treated with either (A) doxorubicin alone (10 *µ*M), (B) doxorubicin (10 *µ*M) and salinomycin (10 *µ*M), (C) doxorubicin (10 *µ*M) and salinomycin (20 *µ*M). (D) pretreatment with doxorubicin alone; (E) pretreatment with doxorubicin and 10 *µ*M salinomycin and (F) pretreatment with doxorubicin and 20 *µ*M salinomycin. The original magnification of all images was x1,800, and the images were captured under the same microscope settings. All data are representative of at least three independent experiments. MDR, multidrug resistance.

The intracellular localization and accumulation of doxorubicin in the MCF-7/MDR cells were observed using laser scanning confocal microscopy. Red fluorescence was observed in the cells (Fig. 2A) following treatment with 10 *µ*M doxorubicin alone for 3 h. The fluorescence observed following treatment with the combination of either 10 or 20 *µ*M doxorubicin with salinomycin led to a marginal increase in the accumulation of doxorubicin, and was dependent on the salinomycin concentration (Fig. 2B and C). At 2 h post-removal of doxorubicin and salinomycin, the fluorescence of the cytoplasmic signal in the control cells treated with doxorubicin alone was almost absent following its removal (Fig. 2D), however, the fluorescence remained evident in the cells pretreated with salinomycin (Fig. 2E and F). These results were consistent with the cell viability data, indicating that salinomycin induced the efflux of doxorubicin from the cells.

### Salinomycin regulates the cellular uptake and efflux of doxorubicin in MCF-7/MDR cells

**Figure 3:**
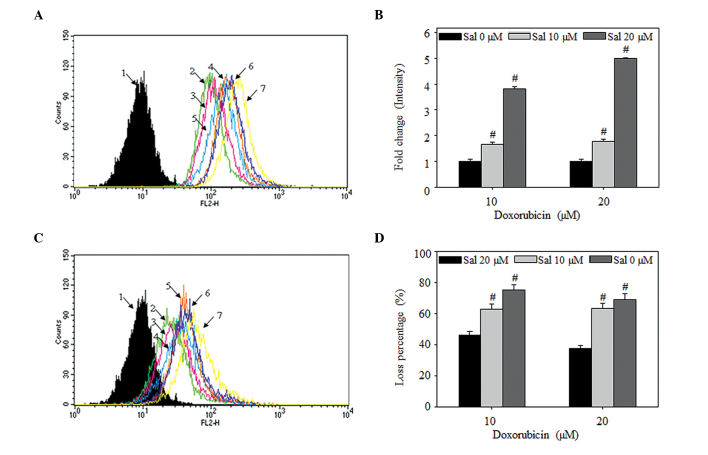
Salinomycin regulates the uptake and efflux of doxorubicin in MCF-7/MDR cells. (A and B) Effects of salinomycin on the accumulation of doxorubicin. The MCF-7/MDR cells were treated with doxorubicin, alone or in combination with salinomycin (10 or 20 *µ*M) for 3 h. (C and D) Effects of salinomycin on the efflux of doxorubicin. The cells were removed from exposure to doxorubicin, but not salinomycin, for 2 h. (A and C) Flow cytometric analysis. 1, untreated; 2, doxorubicin (10 *µ*M); 3, doxorubicin (10 *µ*M) + salinomycin (10 *µ*M); 4, doxorubicin (10 *µ*M) + salinomycin (20 *µ*M); 5, doxorubicin (20 *µ*M); 6, doxorubicin (20 *µ*M) + salinomycin (10 *µ*M); 7, doxorubicin (20 *µ*M) + salinomycin (20 *µ*M). All data are representative of at least three times independent experiment(B) Fold change in the intracellular fluorescent intensity. (D) Percentage loss of intracellular fluorescent intensity.. Data in B and D are expressed as the mean ± standard deviation (^#^P<0.05). MDR, multidrug resistance; Sal, salinomycin.

To determine whether salinomycin mediated the net uptake and efflux of doxorubicin in the MCF-7/MDR cells, the flow cytometric intensities were analyzed. These results demonstrated significant increases, similar to those observed using confocal microscopy, in the net uptake of doxorubicin, and decreased efflux of doxorubicin by salinomycin (Fig. 3A and B). In the case of net uptake, at 3 h post-treatment of the MCF-7/MDR cells with 10 *µ*M doxorubicin with either 10 or 20 *µ*M salinomycin, the fluorescence signal intensities were 1.6- or 3.8-fold higher, respectively, compared with treatment with 10 *µ*M doxorubicin alone. Following treatment with 20 *µ*M doxorubicin with either 10 or 20 *µ*M salinomycin, the intensities were 1.7- and 4.9-fold higher, respectively, compared with treatment with 20 *µ*M doxorubicin alone.

In terms of the efflux of intracellular doxorubicin following removal of the drug from the culture media, the intracellular signal loss was significantly lower in the cells treated with a combination of doxorubicin and salinomycin, compared with that of the cells treated with doxorubicin alone (Fig. 3C and D). Compared with the signal prior to drug removal, the losses in intracellular doxorubicin 2 h post-treatment were 75.1 and 69.3% following 3 h treatment with 10 or 20 *µ*M doxorubicin, respectively. The losses in intracellular doxorubicin were only 63.0 and 46.1% following treatment with 10 or 20 *µ*M salinomycin, compared with those observed following combined treatment with salinomycin and 10 *µ*M doxorubicin, respectively. In the cells treated with a combination of salinomycin and 20 *µ*M doxorubicin, the 63.3% loss of intracellular doxorubicin was reduced to 37.5%. These results demonstrated that salinomycin enhanced doxorubicin-induced cytotoxicity by increasing the influx of doxorubicin and decreasing the efflux of doxorubicin in the MCF-7/MDR cells.

### Salinomycin regulates efflux and influx independently of the gene and protein expression levels of MDR1 and MRP1 in MCF-7/MDR cells

**Figure 4:**
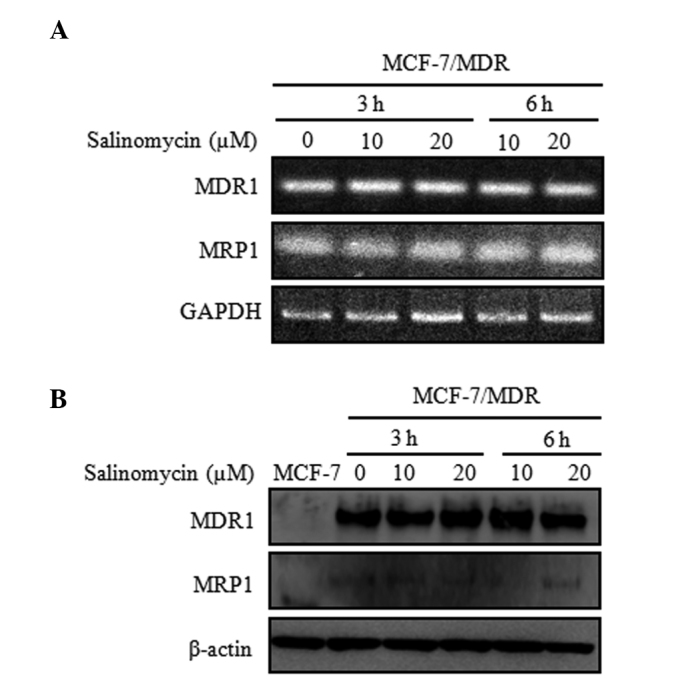
Salinomycin regulates efflux and influx independently of the expression of P-glycoprotein in MCF-7/MDR cells. (A) RT-qPCR and (B) western blot analyses of the expression of MDR-1 and MRP-1 following treatment with salinomycin (10 or 20 *µ*M) for 3 and 6 h. All data are representative of at least three times independent experiments. MDR, multidrug resistance.

To determine the mechanism underlying the reversal effect of salinomycin on the resistance of cancer cells, the present study examined the mRNA and protein expression levels of MDR1 and MRP1 in the MCF-7 and MCF-7/MDR cells. The mRNA and protein levels were estimated using RT-qPCR and western blot analysis. Following treatment with salimycin (10 or 20 *µ*M) for 3 and 6 h, no significant changes were detected in either the mRNA or protein expression levels (Fig. 4), compared with the cells not exposed to salinomycin. However, the MDR1 and MRP1 proteins were not detected in the MCF-7 cells. These results support the suggested that salinomycin did not affect the mRNA and protein expression levels of MDR1 and MRP1 mRNA and protein.

## Discussion

MDR in cancer cells is a significant cause of failure in the chemotherapeutic treatment of several types of cancer (23). MDR involves cross-resistance to unassociated compounds by exposure to an anticancer agent (24). MDR cancer cells often exhibit elevated abilities of increased efflux (ATP-dependent efflux pumps) or decreased influx. The development of MDR is mediated by different mechanisms, and conventional resistance to anticancer drugs has been linked predominantly to the overexpression of ABC transporters, including P-gp, multi-drug resistance-associated protein-1 and the breast cancer resistance protein (12).

Doxorubicin is an anthracycline antibiotic and is commonly used as an effective agent inthe treatment of several types of cancer, including bladder, breast and stomach cancer and multiple myeloma (25). Notably, the resistance of drugs, including doxorubicin, cisplatin and vinblastine, represents a major obstacle in the successful treatment of MDR cancer (26). There have been several reports on the use of doxorubicin in combination with certain other drugs, including fluorouracil and cyclophosphamide (27). The identification of novel combination drugs, which correlate with treatment response, has the potential to improve the success of MDR cancer therapy.

Salinomycin is an ionophore, which is used as a therapeutic antibacterial and coccidiostat (16). A previous study reported that salinomycin causes breast cancer stem cell death, by screening 16,000 different chemical compounds targeting cancer stem cell metastasis and relapse (17). In our previous study, salinomycin was observed to induce apoptosis via the ROS-mediated mitochondrial pathway (19). Other studies have supported these results in variety of cancer cells (23). It has also been demonstrated that salinomycin, as P-gp inhibitors, exhibit potent antiproliferative activity against MDR cancer cells (20).

The present study hypothesized that salinomycin may effectively inhibit binding with P-gp and thus decrease the efflux of anticancer agents from the MDR cancer cells. To investigate this, doxorubicin-resistant MCF-7/MDR cells were treated with doxorubicin, alone or in combination with salinomycin, to assess cell viability. The results revealed the enhancement of salinomycin-mediated cytotoxicity in the MCF-7/MDR cells. This finding was consistent with those of the confocal microscope analysis, in which the doxorubicin fluorescent signals of the salinomycin-treated cells were higher compared with those in the cells treated with doxorubicin alone. In several previous studies, salinomycin has been revealed as a substrate with increased P-gp-dependent transport in MDR cancer cells, as evidenced through drug efflux assays in MDR cancer cell lines (28). Use of a conformational P-gp assay in a previous study provided evidence that the inhibitory effect of salinomycin on P-gp function may be mediated by the induction of ATP transporter conformational change (28). Using flow cytometric analysis, the present study confirmed that salinomycin significantly increased the net cellular uptake and decreased the cellular efflux of doxorubicin. Even 2 h following the removal of doxorubicin and salinomycin, the efflux rate of intracellular doxorubicin remained. Another important feature of salinomycin is that it facilitates bidirectional ion flux through the lipid barrier of membranes, acting as channel blockers to inhibit cell proliferation (29). A similar competitive mechanism has been observed in the reversal of MDR by P-gp inhibitors, including verapamil and cyclosporine A (30,31). In the present study, an efflux was examined using RT-qPCR and western blot analysis, to determine whether this accumulation effect was directly associated with the gene and protein expression of MDR1 and MRP. The expression levels of MDR-1 and MRP-1 were not altered at either the mRNA or protein levels in the cells treated with salinomycin. These results indicated that salinomycin was mediated by its ability to promote the increased uptake and decreased efflux of doxorubicin in the MCF-7/MDR cells.

In analyzing the resistance of MCF-7/MDR cells treated with doxorubicin, the present study demonstrated that salinomycin was a significant inhibitor, with a reversal effect on the resistance of cancer cells. When used in combination with chemotherapeutic agents in MDR cancer, salinomycin may have beneficial therapeutic effects.
